# All-passive pixel super-resolution of time-stretch imaging

**DOI:** 10.1038/srep44608

**Published:** 2017-03-17

**Authors:** Antony C. S. Chan, Ho-Cheung Ng, Sharat C. V. Bogaraju, Hayden K. H. So, Edmund Y. Lam, Kevin K. Tsia

**Affiliations:** 1 Department of Electrical and Electronic Engineering, the University of Hong Kong, Pokfulam, Hong Kong

## Abstract

Based on image encoding in a serial-temporal format, optical time-stretch imaging entails a stringent requirement of state-of-the-art fast data acquisition unit in order to preserve high image resolution at an ultrahigh frame rate — hampering the widespread utilities of such technology. Here, we propose a pixel super-resolution (pixel-SR) technique tailored for time-stretch imaging that preserves pixel resolution at a relaxed sampling rate. It harnesses the subpixel shifts between image frames inherently introduced by asynchronous digital sampling of the continuous time-stretch imaging process. Precise pixel registration is thus accomplished without any active opto-mechanical subpixel-shift control or other additional hardware. Here, we present the experimental pixel-SR image reconstruction pipeline that restores high-resolution time-stretch images of microparticles and biological cells (phytoplankton) at a relaxed sampling rate (≈2–5 GSa/s)—more than four times lower than the originally required readout rate (20 GSa/s) — is thus effective for high-throughput label-free, morphology-based cellular classification down to single-cell precision. Upon integration with the high-throughput image processing technology, this pixel-SR time-stretch imaging technique represents a cost-effective and practical solution for large scale cell-based phenotypic screening in biomedical diagnosis and machine vision for quality control in manufacturing.

High-speed optical imaging with the temporal resolution reaching the nanosecond or even picosecond regime is a potent tool to unravel ultrafast dynamical processes studied in a wide range of disciplines^1^,^2^,^3^,^4^,^5^. Among all techniques, optical time-stretch imaging not only can achieve an ultrafast imaging rate of MHz-GHz, but also allow continuous operation in real time. This combined feature makes it unique for ultrahigh-throughput monitoring and screening applications, ranging from barcode recognition and web-inspection in industrial manufacturing^6^ to imaging cytometry in life sciences and clinical diagnosis^7^. Nevertheless, a key challenge of time-stretch imaging limiting its widespread utility is that the spatial resolution is very often compromised at the ultrafast imaging rate. This constraint stems from its image encoding principle that relies on real-time wavelength-to-time conversion of spectrally-encoded waveform, through group velocity dispersion (GVD), to capture image with a single-pixel photodetector. In order to guarantee high spatial resolution that is ultimately determined by the diffraction limit, two interrelated features have to be considered. First, sufficiently high GVD in a dispersive medium (≈1 ns nm^−1^ at the wavelengths of 1–1.5 μm) is needed to ensure the time-stretched waveform to be the replica of the image-encoded spectrum. Second, time-stretch imaging inevitably requires the electronic digitizer with an ultrahigh sampling rate (≥40 GSa/s) in order to resolve the time-stretched waveform. To avoid using these state-of-the-art digitizers, which incur prohibitively high cost, the common strategy is to further stretch the spectrally-encoded waveform with an even higher GVD such that the encoded image can be resolved by the cost-effective, lower-bandwidth digitizers. However, as governed by the Kramers-Kronig relations, high GVD comes at the expense of high optical attenuation that deteriorates the signal-to-noise ratio (SNR) of the images^8^. Although optical amplification can mitigate the dispersive loss, progressively higher amplifier gain results in excessive amplifier noise, which in turn degrades the SNR. To combat against the nonlinear signal distortion and amplifier noise, it also necessitates careful designs of multiple and cascaded amplifiers that complicate the system architecture. Even worse, achieving high GVD-to-loss ratio becomes increasingly difficult as the operation wavelengths move from the telecommunication band to the shorter-wavelength window, which is favourable for biomedical applications, not to mention the benefit of higher diffraction-limited resolution at the shorter wavelengths. This technical constraint of GVD explains that the overall space-to-time conversion achieved in time-stretch imaging is generally limited to few tens of picoseconds (or less) per resolvable image point. As a consequence, it is common that the sampling rate of the digitizer, i.e. the effective spatial pixel size, is the limiting factor of the spatial resolution in time-stretch imaging, especially in the regime of high analog bandwidth (beyond 1 GHz). In other words, the time-stretch image is easily affected by aliasing if sampled at a lower rate.

To address this challenge, we demonstrate a pixel super-resolution (pixel-SR) technique for enhancing the time-stretch image resolution while maintaining the ultrafast imaging rate. It is possible because high-resolution (HR) image information can be restored from multiple subpixel-shifted, low-resolution (LR) time-stretch images captured by a lower sampling rate. Previously, we demonstrated that subpixel-shifted time-stretch image signal can be recorded in real time by pulse-synchronized beam deflection with the acousto-optic beam deflector (AOD)^9^. However, it requires sophisticated synchronization control for precise sub-pixel registration at an ultrafast rate. It is also viable to perform subpixel-shifted time-stretch image capture by time-interleaving multiple commercial-grade digitizers (TiADC)^10^,^11^. Despite its general availability, this approach is prone to inter-channel timing and attenuation mismatch errors, which degrade the system dynamic range and SNR. It has also been demonstrated that the sampling rate can be effectively doubled by optical replication of spectrally-encoded pulses at a precise time delay^12^. This approach, however, requires high-end test equipment for timing calibration, and is not easily scalable to achieve high resolution gain.

In view of these limitations of the existing techniques, it is thus of great value if a *passive* subpixel-shift scheme using a single commercial-grade digitizer at a lower sampling rate of 1–10 GSa/s can be realized for time-stretch imaging. Here, we propose a simple strategy to allow time-interleaved measurements by the inherent sampling clock drifting because the digitizer sampling clock is unlocked from the pulse repetition frequency of the pulsed laser source. By harnessing this effect at a lower sampling rate, we are able to extract multiple LR time-stretch line-scans, each of which is subpixel-shifted at the precision of tens of picoseconds. This technique resembles the concept of equivalent time sampling adopted in high-end sampling oscilloscope^13^,^14^. In this paper, we demonstrate that the pixel-SR technique is able to reconstruct the HR time-stretch images at an equivalent sampling rate of 20 GSa/s, from the LR images captured at 5 GSa/s. In the context of imaging flow cytometry applications, we also demonstrate that pixel-SR facilitates the morphological classification of biological cells (phytoplankton). Unlike any classical pixel-SR imaging techniques, our method does not require any additional hardware for controlled subpixel-shift motion (e.g. detector translation^15^,^16^ illumination beam steering^17^,^18^), or complex image pixel registration algorithms for uncontrolled motions^19^,^20^, thanks to the highly precise pixel drifting. Therefore, this pixel-SR technique is in principle applicable to all variants of time-stretch imaging systems, including quantitative phase time-stretch imaging^21^,^22^,^23^.

## General Concepts

**Figure 1:**
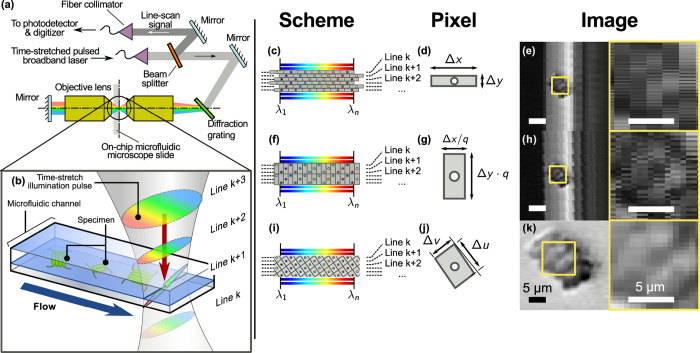
Imaging flow cytometry setup with optical time-stretch capability. (**a**) Imaging flow cytometry setup with optical time-stretch capability; (**b**) illustration of fast-axis scanning by spectral-encoding illumination and slow-axis scanning by ultrafast microfluidic flow; (**c**–**e**) conventional image restoration by aligning the time-stretch line-scans, but disregarding the actual proximity of sampled points in neighboring line scans. (**f**–**h**) Interleaving multiple line-scans can resolve the high bandwidth time-stretch temporal waveform along the fast axis. Both methods give rise to highly elongated pixels with aliasing along the fast axis and the slow axis respectively. (**i**–**k**) Two-dimensional re-sampling utilizes relative subpixel drift *δx* of neighboring line scans to interpolate from the same data. Even though the pixel area in panel (**g**) is the same as that in panel (d), the spatial resolution improves along the fast axis after interpolation. Insets: Zoom-in views of the restored optical time-stretch image.

We consider the most common form of time-stretch imaging that has been proven in a broad range of applications, from flow cytometry to surface inspection, i.e. on-the-fly line-scan imaging of the specimen [Fig. 1(a,b)]. In this scenario, the pulsed and one-dimensional (1D) spectral shower illumination performs spectrally-encoded line-scanning of the unidirectional motion of the specimen, e.g. biological cells in microfluidic flow (see Methods). The two-dimensional (2D) image is reconstructed by digitally stacking the spectrally-encoded and time-stretched waveforms, so that the fast axis of the resultant 2D image is the spectral-encoding direction, and the slow axis corresponds to the flow direction [Fig. 1(c)].

The pixel resolution along the flow direction (slow axis) is the product of linear flow speed *v*_*y*_ and the laser pulse repetition rate *F*, i.e. Δ*y* = *v*_*y*_/*F*. On the other hand, the pixel resolution along the spectral-encoding direction (fast axis) is independently determined by the resolving power of the imaging setup and that of the wavelength-to-time conversion, i.e. Δ*x* = *C*_*x*_(*C*_*t*_* f*)^−1^, where *C*_*x*_ is the wavelength-to-space conversion factor of the spectral encoding setup; *C*_*t*_ is the wavelength-to-time conversion factor of the time-stretch spectrum analyzer; and *f* is the sampling rate of the digitizer. When operated at a low sampling rate, time-stretch imaging of ultrafast flow generates highly elongated pixels [Fig. 1(d)] that easily result in image aliasing [Fig. 1(e)]. We find that the aspect ratio of the original LR image pixel, defined as


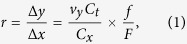


is as small as in the order of 10^−2^ in typical time-stretch imaging configuration (see the parameters described in: Methods). Ideally, if the sampling clock frequency *f* of the digitizer is locked to the laser pulse repetition rate *F*, the line scans will align along the slow axis. In practice, the average number of pixels per line scan (=*f*/*F*) is not an integer. The line scan appears to “drift” along the fast axis, and hence the image appears to be highly warped especially at low sampling rate [Supplementary Fig. S1(b)]. Specifically, as the sampling rate *f* is unlocked from the laser pulse repetition rate *F*, pixel drift between adjacent time-stretch line-scans is observed, and can be expressed as


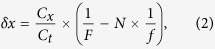


where integer *N* is the number of pixels per line scan rounded off to the nearest integer. It can be shown that |*δx*| ≤ Δ*x*/2. The warp angle is thus given as tan*θ* = *δx*/Δ*y*, as illustrated in Supplementary Fig. S1(b). A common and straightforward approach to dewarp the image is to realign the digitally up-sampled line-scans. However, this would, as shown later, result in image aliasing and artefact that are particularly severe at the lower sampling rate. Furthermore, digital up-sampling of individual line scans does not provide additional image information and thus does not improve resolution along the fast axis. An alternative approach is to interleave multiple line scans to resolve the high bandwidth 1D temporal waveform [Fig. 1(f–h)]. It is commonly known as equivalent time sampling^13^,^14^. However, fusion of multiple line-scans comes with the reduction of pixel resolution along the slow axis, which also introduces image aliasing.

We propose a pixel-SR strategy to harness this warping effect for creating the relative “subpixel shift” on both the fast and slow axes, and thus restoring a high-resolution 2D time-stretch image [Fig. 1(i–k)]. We first register the exact warp angle *θ* of the 2D grid [Supplementary Fig. S1(e)]. It takes advantage of the non-uniform illumination background of the line-scan (mapped from the laser spectrum) as the reference, thanks to the superior shot-to-shot spectral stability offered by the broadband mode-locked laser^5^,^24^,^25^,^26^. Based on the balance between the spectral broadening and gain-narrowing in the all-normal dispersion cavity this pulsed laser, which is home-built based on off-the-shelf fiber components, also provide negligible timing jitter (Supplementary Fig. S5). This is an important factor to ensure the precise sub-pixel registration.

The precision of the measured warp angle critically influences the performance of the pixel-SR algorithm^20^,^27^. Next, the illumination background is suppressed by subtracting the intermediate “dewarped” image [Supplementary Fig. S1(c)] with the high-bandwidth 1D reference illumination signal, which is in turn restored by interleaving the first *q* LR time-stretch line-scans [Supplementary Fig. S1(d)]. The 1D interleaving operation is based on a fast shift-and-add algorithm^28^ together with rational number approximation. Finally, the image is denoised and re-sampled into the regular high-resolution grid^29^, thus reveals high-resolution information [Supplementary Fig. S1(e)]. Detailed steps of the complete pixel-SR algorithm are included in the Supplementary information.

Note that interpolation of neighboring line-scans effectively enlarges the pixel size along the slow axis and reduces the effective imaging line-scan rate. As shown in Fig. 1(g), the dimensions of the interpolated pixel along the warped direction are given as


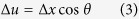



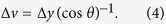


This transform apparently does not resolve problem of aliasing because of the invariant pixel area, i.e. Δ*u*Δ*v* = Δ*x*Δ*y* for all |*θ*| < *π*/2. Nevertheless, when we consider the ratio of pixel size reduction, given as


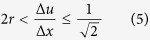



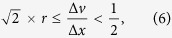


the resolution improvement in the demonstration is particularly significant for highly elongated pixels [Eq. (1) and Fig. 1(d,g)], and a large warping (|tan*θ*| ≥ 1). Both cases are achievable at high repetition rate of ultrafast pulsed laser source (i.e.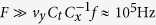), without compromising the overall imaging speed or throughput. Also, the enlarged pixel size along the slow axis after interleaving is still well beyond the optical diffraction limit. Note that the restored image resolution, as opposed to pixel resolution defined in Eqs (3 and 4), is ultimately limited by the optical diffraction limit and the analog bandwidth of the digitizer. In practice, these resolution limits are utilized to construct a matched filter to suppress noise from multiple low-resolution images in our pixel-SR algorithm (see Supplementary information).

## Results

### Pixel-SR time-stretch imaging of phytoplankton

**Figure 2:**
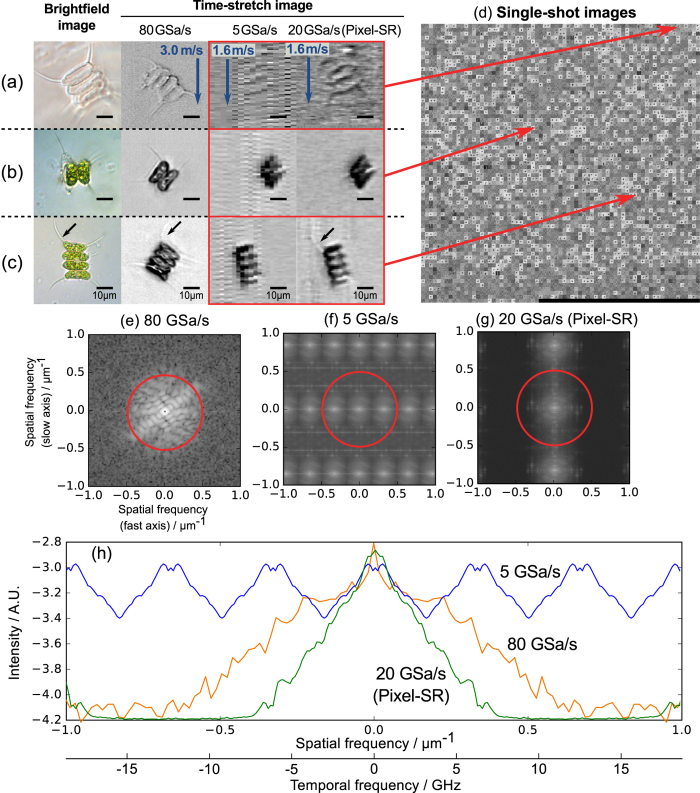
*Scenedesmus* samples captured by pixel-SR time-stretch imaging. Comparison of images in high resolution (80 GSa/s, 0.2 μm/pixel), low resolution (5 GSa/s, 3.6 μm/pixel) and pixel-SR (equivalent to 20 GSa/s, 0.9 μm/pixel) for different cell sub-types: (**a**) discarded exoskeleton; (**b**) colonies with two daughter cells; (**c**) colonies with four daughter cells. Refer to Supplementary Fig. S2 for more examples. (**d**) Image collage of all 5,000 colonies and fragments acquired at 5 GSa/s. (**e**) Corresponding 2D Fourier spectrum of images captured at 80 GSa/s, and (**f**,**g**) Fused 2D Fourier spectra of 5,000 LR and pixel-SR images. The red circle represents the spatial resolution limit at 2 μm, i.e. at around 9.3 GHz temporal bandwidth from the time-stretch microscope. (**h**) 1D profile of the Fourier spectra along the fast axis. Interactive version of panel (d) is available online at http://www.eee.hku.hk/~cschan/deepzoom/.

To demonstrate pixel-SR for ultrafast time-stretch imaging with improved spatial resolution, we chose a class of phytoplankton, *scenedesmus* (Carolina Biological, USA), for its distinct morphological property. *Scenedesmus* is a colony of either two or four daughter cells surrounded by the cell wall of the mother (Fig. 2). Each daughter cell possesses an elongated shape at around 5 μm in diameter along the minor-axis^30^. Therefore, it serves as a model specimen to test resolution enhancement beyond Δ*x* ≈ 2 μm.

In the experiment, individual *scenedesmus* were loaded into the microfluidic channel at an ultrafast linear flow velocity of 1 ms^−1^ to 3 ms^−1^, which was manipulated based on the inertial flow focusing mechanism^31^,^32^. Time-stretch imaging of *scenedesmus* was performed at a line-scan rate of 11.6 MHz, determined by the repetition rate of a home-built mode-locked laser (at center wavelength of 1.06 μm). The wavelength-time mapping was performed by a dispersive fiber module with a GVD of 0.4 ns nm^−1^, which is sufficiently large to satisfy the “far-field” mapping condition, i.e. the spatial resolution is not limited by GVD^8^. The time-stretch waveforms were then digitized by a real-time oscilloscope with adjustable sampling rate between 5 GSa/s and 80 GSa/s. Detailed time-stretch imaging system configuration and experimental parameters are described in Methods. At the highest possible sampling rate (80 GSa/s), the cellular images comes with sharp outline and visible intracellular content [second column, Fig. 2(a–c)]. At a lower sample rate of 5 GSa/s, however, the pixel dimensions become respectively (Δ*x*, Δ*y*) = (3.6 μm, 0.18 μm). As the diffraction limited resolution is estimated to be ≈2 μm, the cell images captured at such a low sampling rate become highly aliased [third column, Fig. 2(a–c)].

Together with the fact that the sampling clock was unlocked from the laser pulse frequency, resolution enhancement by pixel-SR, i.e. to achieve a pixel size smaller than Δ*x*, can thus be adopted in this scenario. To support the above argument, we estimate the theoretical resolution improvement in this experimental setting. From Eq. (2), the relative pixel drifting is known to be roughly *δx* = −1.8 μm. The warping of the 2D grid is given as tan*θ* ≈ −10. Therefore, the pixel-SR scheme can theoretically achieve a pixel resolution enhancement of (cos*θ*)^−1^ ≈ 10 times. It should be cautious that the spatial resolution of the pixel-SR time-stretch imaging is ultimately bounded by diffraction-limited resolution, and the analog bandwidth of the ADC. We also perform a Fourier analysis to investigate the spatial resolution improvement with the pixel-SR method. Based on an image of scenedesmus captured at 80 GSa/s, it can be inferred that the cut-off frequency of the Fourier spectrum is approximately 0.5 μm^−1^ (or equivalently a signal bandwidth of ≈10 GHz) corresponding to an optical resolution of ≈2 μm [Fig. 2(e)]. In the case of the LR image captured at the sampling rate of 5 GSa/s or equivalent pixel size of 3.6 μm, the 2D Fourier spectrum [Fig. 2(f)] is corrupted by signal aliasing due to limited Nyquist bandwidth limit at 2.5 GHz (or spatial frequency of ≈0.13 μm^−1^). To restore the best optical resolution, it would be ideal in this case to choose the number of sub-pixels to be at least 4, i.e. the pixel size of the HR image ≈0.9 μm. This corresponds to an effective sampling rate of 20 GSa/s. The detailed steps related to pixel size selection can be referred to Supplementary Information. However, we note that the restored image resolution is incidentally limited by the built-in signal conditioning filter, also at ≈10 GHz cut-off frequency in the oscilloscope, which could potentially distort the high-frequency information of the image. Nevertheless, with the pixel-SR method, the lost information in the LR image can be restored up to ≈6 GHz [Fig. 2(e,f,h)], i.e. spatial frequency of around 0.3 μm^−1^ — clearly demonstrating the resolution improvement effect of the pixel-SR technique.

The warped grid is subsequently re-sampled to a regular rectangular grid at a pixel dimensions of 0.9 μm × 0.9 μm. The value of the warp angle *θ* is further refined by computational optimization (see Supplementary information) to ensure accurate pixel registration^20^. A spatial averaging filter is constructed to match the estimated optical diffraction limit and electronic filter bandwidth to average out the excess measurements and to suppress noise. The restored pixel-SR images of the corresponding cell types are shown in Fig. 2 and Supplementary Fig. S2. The individual daughter cells in the *scenedesmus* colonies are now clear of aliasing artifacts and noise. Specifically, the hair protruding at the cell body, that is otherwise missing in LR time-stretch image, is now visible in the restored HR image. Note that such information cannot be retrieved simply by interpolation of the LR image — demonstrating the key concept of pixel-SR technique, i.e. combining the non-redundant information in multiple LR frames to construct the HR image.

### Morphological classification of phytoplankton

Detailed spatial information of the cells (i.e. size, shape and sub-cellular texture) can be exploited as the effective biomarkers for revealing cell types, cell states and their respective functions^3^,^7^. Furthermore, such morphological information of cells can readily be visualized and analyzed by label-free optical imaging, i.e. without the concern of cytotoxicity and photobleaching introduced by the fluorescence labelling, not to mention the costly labelling and laborious specimen preparation work. To this end, taking advantage of HR image restoration, pixel-SR time-stretch imaging is particularly useful to enable label-free, high-throughput cellular classification and analysis based on the morphological features, that is not possible with standard flow cytometry. Here, we performed classification of sub-types of *scenedesmus (n* = 5,000) imaged by our optofluidic pixel-SR time-stretch imaging system (sampled at 5 GSa/s). The images of individual colonies are reconstructed by pixel-SR algorithm. Next, the images are computationally screened with a brightness threshold, and then measured by a collection of label-free metrics. The highly parallelized image processing and analysis procedures are performed on the high-performance computing cluster (see Methods and Supplementary Fig. S3). Specifically, we aim at proving the capability of classification of two-daughter colonies and four-daughter colonies based on the label-free pixel-SR images. Since the two variants belong to the same species, they serve as the relevant test subjects for label-free morphology-based cell classification.

**Figure 3:**
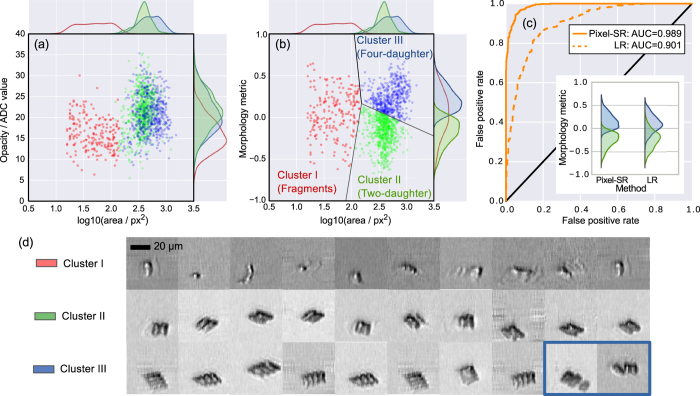
Classification of *scenedesmus* samples based on opacity, area, and morphology. (**a**) Bulk metrics, i.e. opacity and area, are computed from time-stretch images restored by pixel-SR. (**b**) Classification is improved due to the morphology metric computed from high resolution image. The histograms in (**a**) and (**b**) shows the projections of the different clusters onto the axes of opacity, area, and morphology metric. (**c**) Receiver operating characteristic (ROC) curve showing the classification performance with and without pixel-SR based on the morphology metric. The inset shows the reduced overlap between cluster II and III with the pixel-SR method. (**d**) HR image of the cell samples selected from the corresponding clusters. (Bottom right, highlighted) The aggregates of smaller colonies are mis-classified as four-daughter colony, but is clearly distinguishable in pixel-SR time-stretch imaging. The 1,368 samples in the scatter plots were pre-screened from the 5,000 pixel-SR image frames with the brightness threshold. Interactive version is available online at http://www.eee.hku.hk/~cschan/scatter_plot/.

We first retrieved two label-free metrics of single cells: opacity and area from the restored pixel-SR frames (see Methods). These spatially-averaged metrics represent the optical density (attenuation) and the physical size of the *scenedesmus* colonies respectively. Based on the scatter plot of the screened samples (*n*′ = 1,368) with the two metrics [Fig. 3(a)], fragments are easily distinguishable from the live cells because they are significantly smaller and more translucent [see also the images in Fig. 3(d)]. Although it was conceived that the size of four-daughter colonies should be roughly double compared with that of the two-daughter colonies, neither the area nor the opacity can be used to separate the two groups, which appear to be highly overlapped in the scatter plot. Clearly, these spatially-averaged metrics (or essentially LR metrics) failed to account for the subtle morphological differences between the two-daughter and four-daughter colonies, both of which exhibit high variability in both the area and the opacity.

Next, the new morphology metric was extracted from the collections of pixel-SR images, and plotted against cell area in the scatter plot [Fig. 3(b)]. We encoded the morphological features of each image into the histogram of oriented gradients (HoG)^33^, which was then projected to the most significant component using principle component analysis (PCA). Essentially, this metric provides a measure of structural complexity of the cell bodies of the *scenedesmus* colonies, and thus produces better cluster separation compared to opacity metric, with the four-daughter colonies distributed at larger morphology values [i.e. Cluster III in Fig. 3(b)] and two-daughter colonies at smaller values [i.e. Cluster II in Fig. 3(b)]. In order to further substantiate the significance of pixel-SR for improving the classification accuracy, we also compare the classification performance based on the morphology metric extracted from the LR images with that from pixel-SR images. Based on the receiver operating characteristic (ROC) analysis, the area-under-curve (AUC) of the pixel-SR case is ≈0.99 which is higher than the that of the LR case, which is only ≈0.9 [Fig. 3(c)] — showing the ability of pixel-SR to achieve better classification result. The corresponding pixel-SR images are also randomly selected from each cluster for visual inspection, the result of which indicates a good agreement between the classified results and the manually identified groups [Fig. 3(d)]. We provide an interactive plot of Fig. 3(b) that reveals the complete gallery of the HR images of each cluster at higher zoom level (accessible online at http://www.eee.hku.hk/~cschan/scatter_plot).

In practice, multiple morphological metrics can be measured from the pixel-SR image to improve classification accuracy^7^,^34^. Owing to the discrete structure of *senedesmus* colonies, it is possible to separate the two sub-types: two-daughter and four-daughter colonies more effectively in a higher-dimensional morphology metric space. For the sake of demonstration, only the first principle component of the morphological feature set, having the largest variance, is computed here as the morphology metric for classification.

We note that the present classification primarily focuses on the two populations, i.e. two-daughter and four-daughter colonies. Nevertheless, we observe that pixel-SR time-stretch images reveal further heterogeneity within the same population of phytoplankton. Specifically, we identify a group of highly translucent fragments with well-defined exoskeleton structures (Supplementary Fig. S2), and some rare aggregates as marked in blue frames [Fig. 3(c) and Supplementary Fig. S2] — again demonstrating the imaging capability of pixel-SR time-stretch imaging for revealing rich morphological information at lower digitizer sampling rate. Inadvertently, these translucent fragments and the aggregates are currently either computationally rejected as noise or mis-classified as four-daughter colonies, introducing selection bias in the classification procedure. In spite of this, the pixel-SR time-stretch images of all these outliers can be clearly identified by manual inspection alone. More significantly, advanced automated non-linear object recognition algorithms, such as artificial neural network for deep learning^7^, can be coupled with the present pixel-SR technique to improve the classification precision and sensitivity.

### Real-time continuous pixel-SR time-stretch imaging of microdroplets with field-programmable gate array (FPGA)

As mentioned earlier, pixel-SR time-stretch imaging offers a practical advantage over direct acquisition at extremely high sampling rate, i.e. at 40 GSa/s or beyond. Ultrafast analog-to-digital conversion demands costly adoption of the state-of-the-art oscilloscopes that are conventionally equipped with limited memory buffers. Not only does it hinder continuous, real-time on-the-fly data storage, but also high-throughput post-processing and analytics. Pixel-SR time-stretch imaging offers an effective approach to address this limitation by capturing the time-stretch images at a lower sampling rate (in the order of 1 GSa/s), yet without compromising the image resolution. More significantly, unlike the use of high-end oscilloscope in the previous experiments, a commercial-grade digitizer at a lower sampling rate can be readily equipped with an FPGA, capable of continuous and reconfigurable streaming of enormous time-stretch image raw data to the distributed computer storage cluster (see Methods). To demonstrate the applicability of pixel-SR to such a high-throughput data processing platform, we performed continuous real-time monitoring of water-in-oil emulsion microdroplet generation in the microfluidic channel device at a linear flow velocity as high as 0.3 ms^−1^ and at the generation throughput of 5,800 Drpplet/s (see Methods). The time-stretch image signal is continuously recorded at the sampling rate of *f* = 3.2 GSa/s.

**Figure 4:**
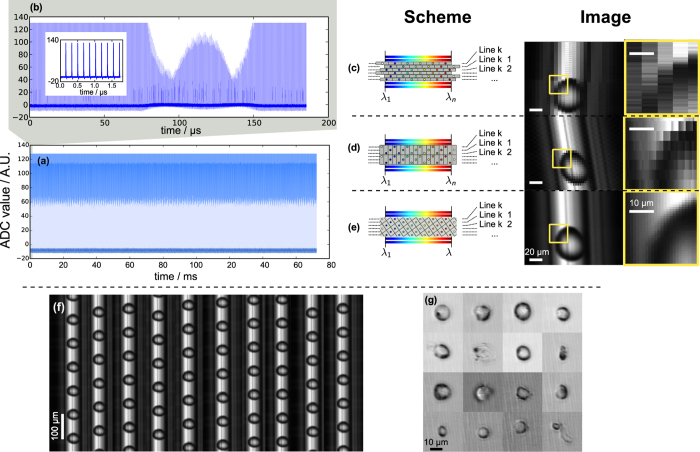
Continuous time-stretch image acquisition of water-in-oil microdroplets and human acute monocytic leukemia (THP-1) cells at 3.2 GSa/s. Serialized time-stretch signal of (**a**) 170 ms duration, and (**b**) the first 200 μs. The inset shows the first 10 line scans of the measurement. Image restoration by (**c**) Pulse-by-pulse alignment; (**d**) One-dimensional equivalent time sampling; (**e**) Pixel super-resolution (pixel-SR) result. The zoomed-in view of the images are also shown in the insets. (**f**) Snapshots of continuous recording of water-emulsion droplet imaging at a regular interval of 10 ms. Totally 978 droplets are captured within the 170 ms duration. (**g**) Snapshots of THP-1 cells.

Similar to the previous observations with the oscilloscope, the raw time-stretch image captured by the commercial-grade digitizer is highly warped because of the timing mismatch between the laser and the digitizer [Fig. 4(b,c)]. It is reminded that the frequently adopted approach is to dewarp the image by aligning the individual line scans. As shown in Fig. 4(c), each line scan is digitally re-sampled to realign the pixels along the slow axis. While this strategy used to work for oversampled time-stretch signal at 16 GHz bandwidth^35^,^36^, it does not perform well at the low sampling rate because of signal aliasing [Fig. 4(c)]. Again, interleaving multiple line-scans comes with the degradation of pixel resolution along the slow axis as shown in Fig. 4(d). In contrast, our pixel SR algorithm is able to restore HR time-stretch image, avoiding aliasing on either fast and slow axes. The resolution improvement is five times the apparent pixel size, i.e. at effective sampling rate of 16 GSa/s and at pixel resolution of 1.1 μm [Fig. 4(e,f)].

Similar results are also achieved by the commercial-grade digitizer and FPGA with biological specimen. Here we flow the human acute monocytic leukemia cells (THP-1) at a flow speed of ≈2 ms^−1^ and the captured time-stretch image signal is captured by the same FPGA platform. Clearly, the outline of THP-1 cells can be restored by our pixel-SR algorithm [Fig. 4(g)].

It is noted that all image restoration procedures are currently done offline. Although only the first 170 ms is captured in this experiment for the sake of demonstration, the maximum number of image pixels in the continuous image capture can be scaled up to the total data capacity of the computer cluster. In our system, each computer node is equipped with the 256 GB hard disk drive [Supplementary Fig. S4(a)]. In principle, our method can enable real-time morphological image capture and object recognition at giga-pixel capacity.

## Discussions

The spatial resolution of ultrafast time-stretch imaging is closely tied with the temporal resolution during data capture, especially the sampling rate of the digitizer. This feature of space-time mapping implies an overwhelming requirement on ultrahigh sampling rate, and thus the state-of-art digitizer in order to avoid image aliasing. Not only does such high-end digitizer incur prohibitive high cost, but it also lacks sufficient memory depth for high-throughput continuous data storage, processing and analytics. These key challenges have been impeding the widespread utility of time-stretch imaging in high-throughput applications, ranging from machine-vision and quality control in industrial manufacturing to single-cell analysis in basic biomedical and clinical diagnosis.

Here, we proposed and demonstrated a pixel-SR technique that could enhance the pixel resolution (i.e. anti-aliasing) of ultrafast time-stretch imaging at the lower sampling rate, which is largely supported by the commercial-grade digitizers. Resembling the concept of equivalent time sampling that is employed high-speed sampling oscilloscopes, our pixel-SR technique harnesses the fact that subpixel shift of consecutive time-stretch line scans is innately generated by the mismatch between laser pulse repetition frequency and sampling frequency — a feature appeared virtually in all types of time-stretch imaging modalities. Therefore, it requires no active synchronized control of illumination or detection for precise sub-pixel shift operation at an ultrafast rate.

It is worth mentioning that compressive sampling can be an alternative solution to combat image aliasing at lower sampling rate, which utilizes pseudo-random illumination patterns to preserve HR information of the image^37^,^38^. The same effect is also achieved by modulating the spectrally-encoded pulse after illumination^39^,^40^. Although around two-order-of-magnitude reduction in digitizer bandwidth has been demonstrated with this technique^38^,^40^, it requires time-consuming iterative process to restore the HR image. In contrast, our method involves one-off 2D image re-sampling in the HR grid, thus produces a much lower computational footprint than the compressed sensing algorithms.

Incidentally, the sub-pixel registration — a key to ensure the robustness of the pixel-SR reconstruction, has been challenging as the sub-pixel shift is very often arbitrary and is further complicated by the motion blur. Thanks to the superior shot-to-shot line-scanning offered by the stable mode-locked laser as well as the motion-blur-free imaging guaranteed by the ultrafast line-scan (i.e. at MHz and beyond), our technique allows high-precision sub-pixel shift estimation. We have demonstrated the strength of this method by enhancing pixel resolution of existing time-stretch imaging flow cytometry setup without additional hardware. In our experiments, we showed that the digitizer sampling rate can be relaxed to 5 GSa/s with our technique from the original 80 GSa/s. Cellular texture, which is otherwise obscured in the LR time-stretch images, can be restored with the pixel-SR algorithm. More importantly, the restored HR time-stretch images enables better classification of biological cell sub-types. Notably, we have also implemented the pixel-SR time-stretch imaging technique with the high-throughput data-acquisition platform based on FPGA at the sampling rate of 3.2 GSa/s. Note that the compatibility of the pixel-SR algorithm to the FPGA is significant, in that the integration of both can represent a cost-effective and practical solution for a wide variety of high-throughput time-stretch imaging applications. While this pixel-SR technique is currently demonstrated in the context of time-stretch imaging, this concept can be generally applicable to any ultrafast line-scan imaging modalities with a single-pixel detector, where the asynchronous sampling is involved in the image data capture.

In this paper, the interpolation algorithm is executed on a single processing core for each 1D time-stretch data segment representing one image frame; the 5,000 image frames of *scenedesmus* colonies (see Methods) are restored independently in multiple cores of the high-performance computing cluster [Supplementary Fig. S4(b)] to achieve a real-time combined data crunching rate of 26.0 MSa/s ≈ 104 framespersecond. Conceptually, the pixel-SR algorithm can be implemented in the graphical processing unit (GPU)^41^,^42^ as a massively parallel routine to increase the data crunching rate by up to two orders-of-magnitude, i.e. in the order of 10 GSa/s. As mentioned before, this algorithm can be programmed in the FPGA for real-time image restoration and classification, further eliminating the back-end computation resources depicted in Supplementary Fig. S4(b). This will be the next stage of our recent work on real-time *in situ* classification^36^.

## Methods

### Optofluidic time-stretch microscopy system

Figure 1(a) shows the schematics of the optofluidic time-stretch microscope with a double-pass configuration^43^. The microfluidic channel is illuminated by a spectrally-encoded pulsed laser beam (center wavelength = 1060 nm; bandwidth = 20 nm). As the biological cells or microparticles travel along the microfluidic channel, a train of time-stretched illumination pulses captures a sequence of line-scans across the cell at a laser pulse repetition rate of *F* = (11.6142 ± 0.0005) MHz. The detection module, which consists of a 12 GHz photodetector (1544-B, Newport) and the 33 GHz-bandwidth digital storage oscilloscope (DSAV334A, Keysight Technologies), then records and digitizes the captured line-scan sequence at the instaneous sampling rate from 5 GSa/s to 80 GSa/s [Fig. 1(b)]. The infinity-corrected microscope objective lens (L-40X, Newport) at numerical aperture of 0.66 and the transmission diffraction grating (WP-1200, Wasatch Photonics) at 1200 groove achieve the wavelength-to-space conversion factor of *C*_*x*_ = 7.1 μm nm^−1^. The wavelength-to-time conversion factor *C*_*t*_ = 400 ps nm^−1^ is achieved with a 5 km long single-mode dispersive fiber (1060-XP, Nufern). The values of both conversion factors are kept fixed in our experiments.

### Imaging flow cytometry protocol

A population of more than 10,000 units of phytoplankton is loaded into the microfluidic channel with a syringe pump (PHD22/2000, Harvard Apparatus) at a linear speed of 1.6 ms^−1^, A polydimethylsiloxane (PDMS) microfluidic channel is designed and fabricated based on ultraviolet (UV) soft-lithography. Detailed fabrication steps has been described in ref. 35. The width (60 μm) and height (30 μm) of the channel are chosen such that the balance between the inertial lift force and the viscous drag force is achieved for manipulating the positions of the individual cells (with the size of ≈5–20 μm) and focusing them in ultrafast flow inside the channel^31^,^32^.

The time-stretch signal is first captured and digitized by the oscilloscope (DSAV334A, Keysight Technologies) of maximum analog bandwidth of 33 GHz. Without changing hardware, the sampling rate of the oscilloscope is down-adjusted from 80.000 GSa/s to 5.000 GSa/s. At lower sampling rate, it is known to possess a signal conditioning filter at 10 GHz cut-off frequency. Because of the limited memory depth of the oscilloscope, the time-stretch waveform is captured in segmented mode, where only 5,000 units are captured during the experiment [Fig. 2(a)]. More examples of pixel-SR images are shown in Supplementary Fig. S2.

The 2D Fourier spectra in Fig. 2(f,g) are obtained by fusing individual power spectrum of 5,000 images of *scenedesmus*. By projecting only the maximum intensity values in individual spectra, the full signal bandwidth at all cellular sizes and orientations can be accounted for.

### Morphological classification methods

For classification of unlabelled cell by morphology, we first attempt to group the cells in terms of cell mass and volume. These metrics are represented by cell opacity and area respectively, both measured from each single-colony image captured from the experiment [Fig. 3(a)]. *Opacity* is computed by taking the average of all pixel values, before subtracting the background pixel value. *Area*, on the other hand, is computed from the rotated rectangular box enclosing the cell in the image. The rectangular box is tightly fitted to the outline of the external exoskeletons of the *scenedesmus*, which in turn is extracted by a brightness threshold filter, to minimize the area. The samples with zero area, i.e. cells/fragments that are nearly transparent, are screened out before the classification. To compute the morphological metrics, the cell bodies of the *scenedesmus* is first cropped, rotated and then scaled with the tightly-fitted rectangular box that is obtained earlier. This step is to ensure that the morphological metric would be invariant to scale, rotation and aspect-ratio of the cell body. A set of histogram of oriented gradients measurements (HoG) is then computed from the intermediate image, which is then projected to the most significant component using principle component analysis (PCA). As mentioned in the discussions, the cell samples are automatically classified into three disjoint clusters by the K-means clustering algorithm. To avoid human bias, initial cluster centroids are generated from random coordinates in the morphological metric versus log10(area) space, as presented in Fig. 3(b). The exact names of the three clusters (i.e. cell fragments, two-daughter colony and four-daughter colony) are later identified by inspecting the pixel-SR images in each cluster.

### Continuous high-throughput imaging of emulsion generation with field-programmable gate array (FPGA)

In Fig. 4, the water-in-oil emulsion microdroplets are generated *in situ* in the PDMS-based microfluidic water injection device^44^,^45^, which is mounted on the imaging flow cytometry setup. The water microdroplets are in laminar flow at a linear speed of 0.3 ms^−1^. The time-stretch signal is digitized by the 3.2000 GSa/s 8-bit analog-to-digital converter (ADC) (a custom design by the Academia Sinica Institute of Astronomy and Astrophysics, Taiwan), and is subsequently distributed in real-time to four computing workstations by a field-programmable gate array (FPGA) (Virtex-6 SX475T, Xilinx^46^) The digital acquisition system is illustrated in Fig. S4(a). Since each workstation is equipped with the 256 GB solid-state hard drive, the system is capable of continuous high-throughput recording in the order of 10^3^ GPixels. For the sake of demonstration, only the first 160 ms is recorded.

## Additional Information

**How to cite this article:** Chan, A. C. S. *et al*. All-passive pixel super-resolution of time-stretch imaging. *Sci. Rep.* **7**, 44608; doi: 10.1038/srep44608 (2017).

**Publisher's note:** Springer Nature remains neutral with regard to jurisdictional claims in published maps and institutional affiliations.

## Supplementary Material

Supplementary Information
