# Supplementary material for: All-passive pixel super-resolution of time-stretch imaging
Source: Sci Rep. 2017 Mar 17;7:44608. doi: 10.1038/srep44608 (PMC5356014; doi:10.1038/srep44608)
Supplement: Supplementary Information [file srep44608-s1.pdf]

All-passive pixel super-resolution of time-stretch imaging: supplementary information

Antony C. S. Chan[1,2], Ho-Cheung Ng[1,3], Sharat C. V. Bogaraju[1,4], Hayden K. H. So[1], Edmund Y. Lam[1], and Kevin K. Tsia[1,*]

[1]Department of Electrical and Electronic Engineering, the University of Hong Kong, Pokfulam, Hong Kong
[2]Current address: Department of Electrical Engineering, California Institute of Technology, Pasadena, California, 91125, USA
[3]Current address: Department of Computing, Imperial College London, London SW7 2AZ, United Kingdom
[4]Current address: Department of Computer Science and Engineering, National Institute of Technology Goa, India
[*]tsia@hku.hk

ABSTRACT

This document provides supplementary information to the "All-passive pixel super-resolution of time-stretch imaging", **7**, 44608 (2017).

Supplementary information

General framework of pixel super-resolution algorithm

The optofluidic time-stretch imaging process is modelled as

$$g(x,y) = h_1(x,y) * f(x,y) + I_B(x) \tag{1}$$

$$I(t) = h_2(t) * S[g(x + y\tan\theta, y)] + n(t), \tag{2}$$

where $I(t)$ is the distorted low-resolution measurement of the object $f(x,y)$ at the presence of measurement noise $n(t)$; and $g(x,y)$ is the intermediate image of the object illuminated by the spectrally-encoded line beam $I_B(x)$. Functions $h_1(x,y)$ and $h_2(t)$ correspond to the 2D point spread function (PSF) of the optical system and the 1D signal pre-conditioning filter of the digitizer respectively. The warp angle θ accounts for the image distortion brought by the asynchronous sampling of the time-stretch pulse train. The serialization operator $S(\cdot)$, representing the line-scan process, uniquely maps each spatial coordinates (x,y) to time t. The continuous 1D signal $I(t)$ is subsequently sampled by the digitizer. Here, we seek to restore the object $f(x,y)$ from the time-stretch measurement $I(t)$ with the pixel-SR algorithm.

Step 1: Signal de-serialization. Prior to high-resolution image restoration, each input 1D signal $I(t)$ [Supplementary Fig. 1(a)] is first de-serialized to form an intermediate image $I(x,y)$, which doesn't modify the digital samples [Supplementary Fig. 1(b)]. By re-arranging the terms in Eqs. (1–2), the image corruption model can be represented as

$$I(x,y) = W_\theta[h(x,y) * f(x,y) + I'_B(x)] + n(x,y), \tag{3}$$

where $h(x,y) = h_1(x,y) * h_1(t = C_t C_x^{-1} x)$; and $I'_B(x) = h_2(x) * I_B(x)$. The image warp transform operator $W_\theta[\cdot]$ maps the spatial coordinates such that $(x,y) \mapsto (x + y\tan\theta, y)$. In the following paragraphs, we describe the methods to estimate the image distortion parameters from the captured data [i.e. $W_\theta(\cdot)$, and $I'_B(x)$], and subsequently restore the object $f(x,y)$ by denoised non-uniform interpolation.

Step 2: Pixel registration by image warp estimation. The performance of pixel-SR is highly sensitive to errors in pixel registration. The initial value of the relative pixel drift δx can be obtained from the specification of the pulsed laser and the digitizer. However, its precise value can only be estimated from the captured data because the laser cavity length varies in accordance with ambient temperature and mechanical perturbation. In our approach, we achieve accurate pixel registration by optimizing background suppression. Compared to the moving object $f(x,y)$ that contributes to varying spectral shape in the time-stretch line scans, the laser spectral shape $I'_B(x)$ is highly stable from pulse to pulse, and appears as straight bands in the background of the captured raw data. Owing to the presence of pixel drift, the line scans are warped at an angle θ [Supplementary Fig. 1(b)]. This warping needs to be compensated for accurate extraction of the lasing spectrum, evaluated as

$$I'_B(x)|_\theta \approx \frac{1}{M\Delta y} \int_0^{M\Delta y} W_\theta^{-1}[I(x,y)]\, dy, \tag{4}$$

where M is the number of line scans of the warped image I. Ideally, a "clean" foreground [Supplementary Fig. 1(f)] can be obtained by direct subtraction of the lasing background $I_B(x)$ from the dewarped image $W_\theta^{-1}[I(x,y)]$. Error in the value of the warp angle θ induces distortions in the estimated illumination background $I_B(x)$, thus results in band-like artefacts superimposed onto the foreground object. However, this

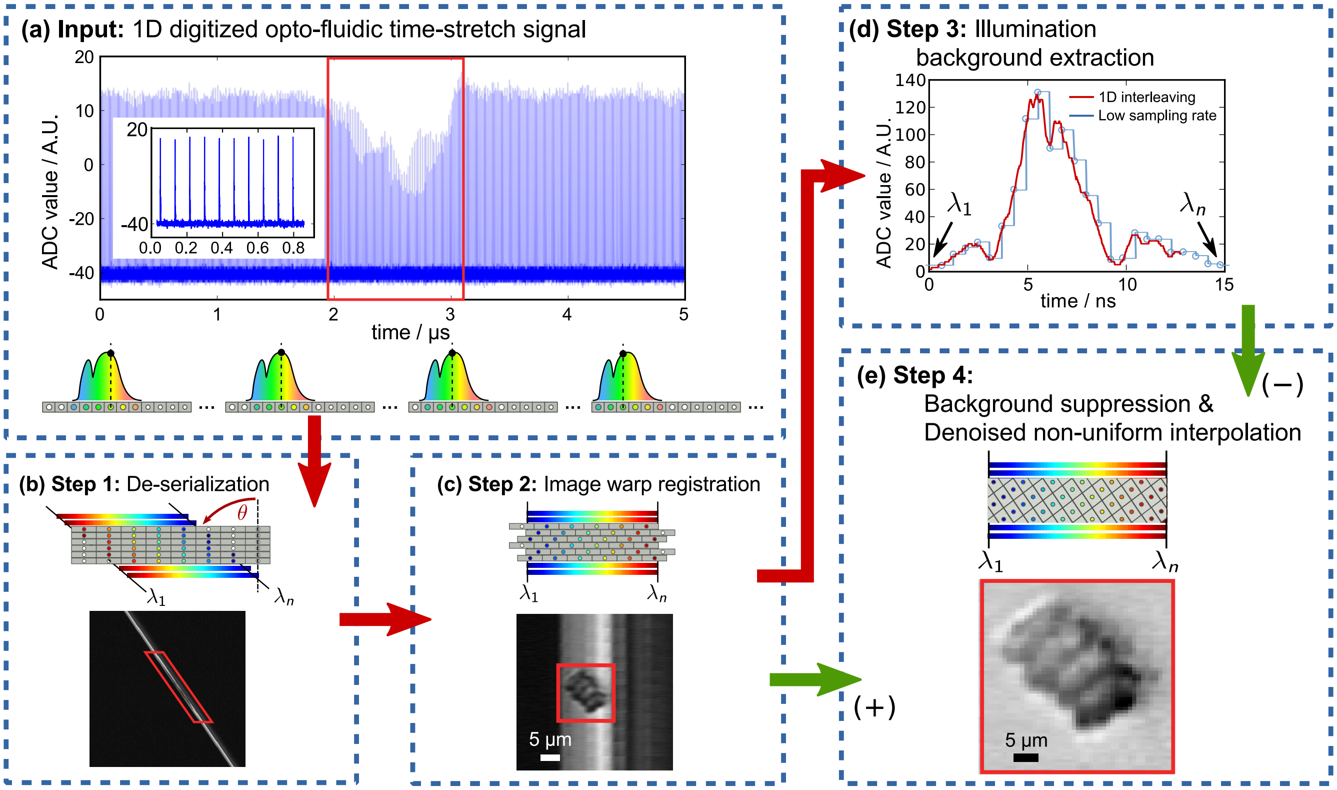

Figure 1. Flow chart of the pixel-SR algorithm for optofluidic time-stretch microscopy.
(a) Serialized time-stretch signal of water emulsion droplet. The inset shows the first 10 line scans of the measurement. (b) De-serializing the time stretch signal, showing the high warp angle θ due to pixel drift. (c) With alignment of the illumination pulse, the warp angle θ can be precisely estimated, so does the exact pixel coordinates in space. (d) Illumination background extraction by 1D equivalent time sampling of the first q line scans.
(e) Denoised non-uniform interpolation of the aligned measurement.

property can be exploited to obtain an accurate value $\hat{\theta}$ by maximizing the "cleaniness" of the extracted foreground, i.e. by minimizing the squared residual in the foreground, expressed as

$$\hat{\theta} = \arg\min_{\theta} \int_0^{N\Delta x} \int_0^{M\Delta y} \left[W_\theta^{-1}[I(x,y)] - I'_B(x)|_\theta \right]^2 dy\, dx, \tag{5}$$

where the integer N is the number of pixels of each line scan. Supplementary Figure 1(c) depicts such image warp registration process. The accuracy of this pixel registration approach is fundamentally limited by the "decorrelation distance" of the laser spectrum, or alternatively named the spectral coherence of the time-stretched illumination pulse, i.e. $\epsilon[\tan\theta] < C_x \delta\lambda / (M\Delta y)$.

The estimated warp angle θ is then utilized to compute the spatial coordinates of all pixels in the low-resolution line scans $I(x,y)$, as shown in Supplementary Fig. 1(c). The registered pixel coordinates and the corresponding pixel value (x_j, y_j, I_j) are then indexed in the k-dimensional (K-D) tree structure[1] for efficient searching.

Step 3: Illumination background extraction. The undulated laser spectrum extracted in Eq. (4), i.e. $I'_B(x)|_{\theta=\hat{\theta}}$, is also aliased; it must be restored to further suppress the illumination background. This problem can be solved by interleaving the first several line scans, in which the object is absent [Supplementary Fig. 1(d)]. A fast shift-and-add algorithm[2] is implemented to interleave the first q low-resolution time-stretch pulses into the high-resolution 1D grid. The time-stretch pulses are zero-filled and shifted before adding up the signals. To enable fast pixelized operations, the relative shift of the k-th pulse ($k \leq q$) is rounded to the multiple of $\Delta x / q$. It is desirable for q to be large to achieve a higher pixel registration. However, time-interleaving of multiple pulses reduces the effective imaging line-scan rate. In other words, the effective pixel size along the slow axis ($q\Delta y$) must be smaller than the optical diffraction limit to avoid image aliasing. The optimization criteria of q is formulated as

$$\min_{0<q<r/\Delta y} \left| \frac{f}{F} - \left(N + \frac{p}{q} \right) \right|, \tag{6}$$

where p,q are integers; integer N is the number of pixels per line scan rounded off to the nearest integer; r is the diffraction limit of the optical microscopy system. Mathematically, this problem is equivalent to the rational number approximation, where the solution is the truncated continued fraction of f/F computed from the Euclidean algorithm[3]. The relative subpixel shift of the k-th pulse ($k \leq q$) is thus determined as $d_k = [p(k-1) \bmod q]/q \times \Delta x$.

This time-interleaving algorithm is also applied in Fig. 4(d). At the sampling rate of 3.2 GSa/s, the best approximation of f/F is $(275\frac{16}{31})$, i.e. $N = 276$; $p = -15$; and $q = 31$. The low-resolution signals of the time-stretch pulses are thus shifted and added in the following locations:

$$\{d_k\} = \left\{0, \frac{16}{31}\Delta x, \frac{1}{31}\Delta x, \frac{17}{31}\Delta x, \frac{2}{31}\Delta x, \ldots, \frac{15}{31}\Delta x\right\}.$$

The anti-aliased laser spectrum is given as[4]

$$\hat{I}'_B(x) = \sum_{k=1}^{q} I[x,(k-1)\Delta y] \times \text{comb}\left(\frac{x-d_k}{\Delta x}\right), \quad (7)$$

where comb$(\cdot)$ is a train of impulse functions[5].

Step 4: Denoised non-uniform interpolation. Pixel-SR restoration of the object $f(x,y)$ can be obtained in two stages: non-uniform interpolation and image denoising[6,7]. For higher computational efficiency, the above two stages are performed at once by utilizing the value of the denoising filter as the weights in the interpolation process [Supplementary Fig. 1(e)]. Our objective is to restore $f(x,y)$ from Eq. 3 by minimizing the noise $n(x,y)$, i.e.

$$\begin{aligned}\hat{f}(x,y) &= \arg\min_f \int_0^{M\Delta y}\int_0^{N\Delta x} [n(x,y)]^2\,dx\,dy \\ &= \arg\min_f \int_0^{M\Delta y}\int_0^{N\Delta x} [I(x,y) - \\ &\quad W_\theta\{h(x,y) * f(x,y) + \hat{I}'_B(x)\}]^2\,dx\,dy \quad (8)\end{aligned}$$

For a digital signal I' and the discrete image $\mathbf{f} = \{f_i : f_i = f(x_i,y_i)\}$, Eq. (8) can be rewritten as

$$\hat{\mathrm{f}} = \arg\min_{\mathbf{f}} \|\mathrm{I}' - \mathbf{Hf}\|_2^2, \quad (9)$$

where $\mathbf{W}_\theta$ and $\mathbf{H}$ are the matrix representation of the operator $W_\theta[\cdot]$ and the convolution kernel $h(x,y)$ respectively; and $\mathrm{I}' = \mathbf{W}_\theta^{-1}\mathrm{I} - \hat{\mathrm{I}}'_B$ is the dewarped, background-suppressed time-stretch signal from Step 3. Solving Eq. (9) directly is not feasible for ultrafast imaging application, not only because of the sheer size of matrix $\mathbf{H}$, but also of the potential noise amplification effect of the ill-conditioned problem[8]. In practice, Eq. (8) can be computed more efficiently by exploiting that fact that the kernel $h(x,y)$ is sparse. That is, the target pixel area of the high-resolution image $f(x,y)$ is set to be just slightly smaller than the area of the 2D convolution kernel $h(x,y)$, such that the kernel $h(x,y)$ at the location of i-th pixel (x_i,y_i), which corresponds to the i-th column of matrix $\mathbf{H}$, is only weakly correlated to that of the neighbouring pixels, i.e. $|\mathrm{h}_i^T\mathrm{h}_j| \ll |\mathrm{h}_i^T\mathrm{h}_i|$ for all $j \neq i$. This pixel size selection also achieves critical sampling of the high-resolution image $f(x,y)$. Hence, the minimizer in Eq. (9) can now be approximated as

$$\begin{aligned}\min_{\mathbf{f}} \|\mathrm{I}' - \mathbf{Hf}\|_2^2 &= \min_{\mathbf{f}} \left\|\mathrm{I}' - \sum_{j=1}^{L}\mathrm{h}_i f_i\right\|_2^2 \\ &\approx \min_{\mathbf{f}} \sum_{i=1}^{L} \|\mathrm{I}' - \mathrm{h}_i f_i\|_2^2 - (L-1)\|I'\|_2^2 \quad (10) \\ &\approx \sum_{i=1}^{L} \min_{f_i} \|\mathrm{I}' - \mathrm{h}_i f_i\|_2^2 - (L-1)\|I'\|_2^2, \quad (11)\end{aligned}$$

where L is the total number of high-resolution pixels of image $f(x,y)$; and h_i is the i-th column of matrix $\mathbf{H}$. For the i-th element of $\mathbf{f}$ at registered coordinates (x_i,y_i), its pixel value is

$$\begin{aligned}\hat{f}_i &\approx \arg\min_{f_i} \|\mathrm{I}' - \mathrm{h}_i f_i\|_2^2 \\ &= \frac{\mathrm{h}_i^T\mathrm{I}'}{|\mathrm{h}_i|} \\ \Rightarrow \hat{f}(x_i,y_i) &\approx \frac{\sum_{j=1}^{MN} h(x_j-x_i,y_j-y_i) \times [I_j - \hat{I}_B(x_j)]}{\sum_{j=1}^{MN}[h(x_j-x_i,y_j-y_i)]^2} \quad (12) \\ &= \frac{\sum_{r_m\leq\sigma} h(x_m-x_i,y_m-y_i) \times [I_m - \hat{I}_B(x_m)]}{\sum_{r_m\leq\sigma}[h(x_m-x_i,y_m-y_i)]^2}, \quad (13)\end{aligned}$$

where pixel value I_m corresponds to the m-th nearest neighbour of the spatial coordinate (x_i,y_i); and distance $r_m = \sqrt{(x_i-x_m)^2 + (y_i-y_m)^2}$. Note that computing Eq. (13) is more efficient than computing Eq. (12) because only the pixel values I_m within the effective radius σ needs to be selected, as depicted in Supplementary Fig. 3. The K-D tree structure, generated earlier in the pixel registration step, is thus utilized for fast searching of neighboring pixels of any given coordinates (x_i,y_i). The kernel $h(x,y)$, that also acts as a denoising filter, is currently approximated as a truncated 2D Gaussian function. The quality of the restored image $\hat{f}(x,y)$ can be further improved by measuring the 2D point spread function (PSF) of the imaging setup and the one dimensional impulse response of the antialiasing filter in the digitizer.

References

1. Maneewongvatana, S. & Mount, D. M. On the efficiency of nearest neighbor searching with data clustered in lower dimensions. In *ICCS: International Conference San Francisco*, 842–851, DOI:10.1007/3-540-45545-0_96 (Springer, Berlin, Heidelberg, 2001).
2. Elad, M. & Hel-Or, Y. A fast super-resolution reconstruction algorithm for pure translational motion and common space-invariant blur. *IEEE Transactions on Image Processing* **10**, 1187–1193, DOI:10.1109/83.935034 (2001).
3. Stillwell, J. *Elements of Number Theory* (Springer New York, 2003).
4. Ben-Ezra, M., Zomet, A. & Nayar, S. Video super-resolution using controlled subpixel detector shifts. *IEEE Transactions on Pattern Analysis and Machine Intelligence* **27**, 977–987, DOI:10.1109/tpami.2005.129 (2005).
5. Bracewell, R. N. *Fourier transform and its applications* (McGraw-Hill, Boston, 2000), 3 edn.
6. Elad, M. & Feuer, A. Restoration of a single super-resolution image from several blurred, noisy, and undersampled measured images. *IEEE Transactions on Image Processing* **6**, 1646–1658, DOI:10.1109/83.650118 (1997).
7. Park, S., Park, M. & Kang, M. Super-resolution image reconstruction: a technical overview. *IEEE Signal Processing Magazine* 21–36, DOI:10.1109/MSP.2003.1203207 (2003).
8. Lam, E. Y. Noise in superresolution reconstruction. *Optics Letters* **28**, 2234–2236, DOI:10.1364/ol.28.002234 (2003).

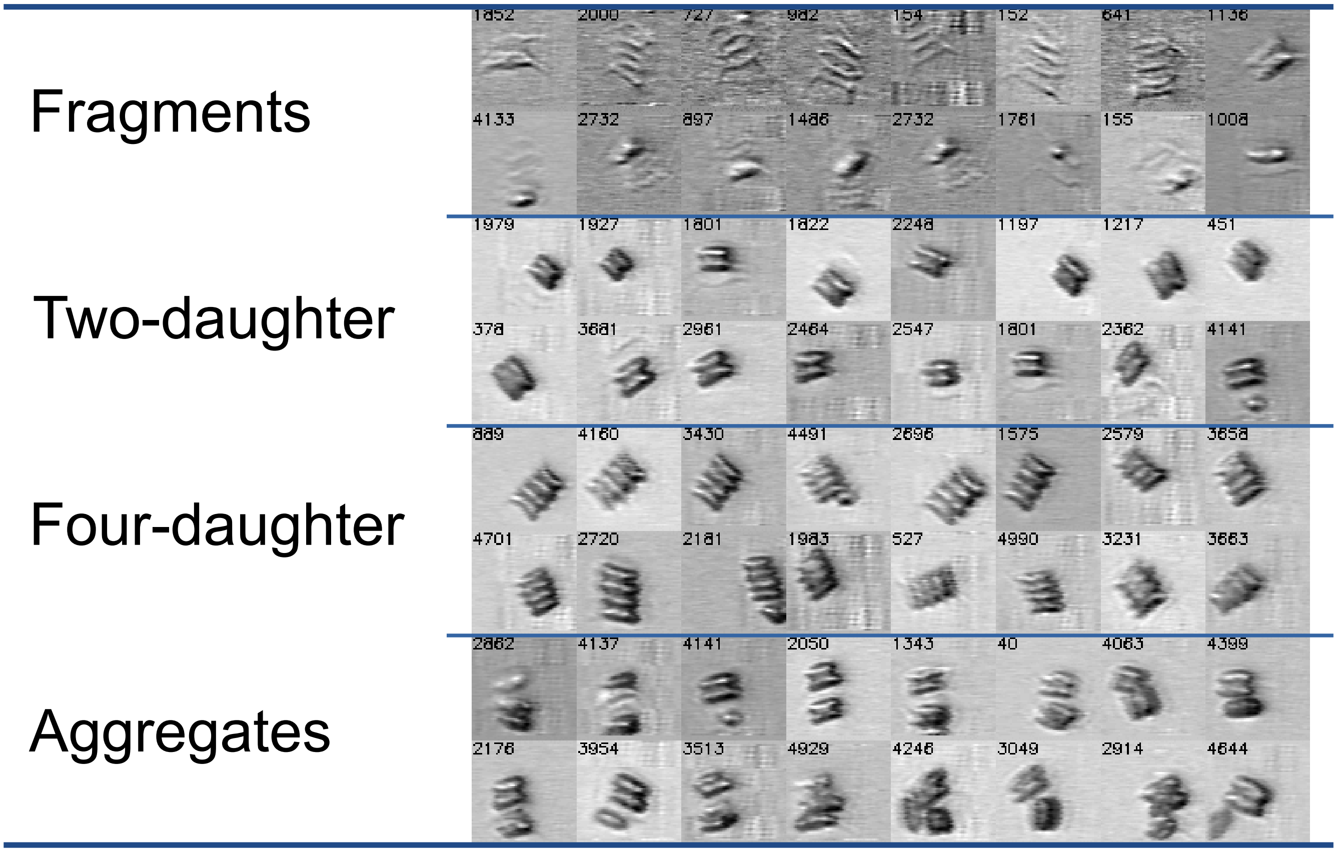

Figure 2. **Further examples of pixel-SR time-stretch image restoration of *scenedesmus*.**
The four-digit number at the top-left corner of each image indicates the *i*-th image frame captured by the optofluidic time-stretch microscope setup. Effective sampling rate: 20 GSa/s. Image scale: 53 µm × 53 µm.

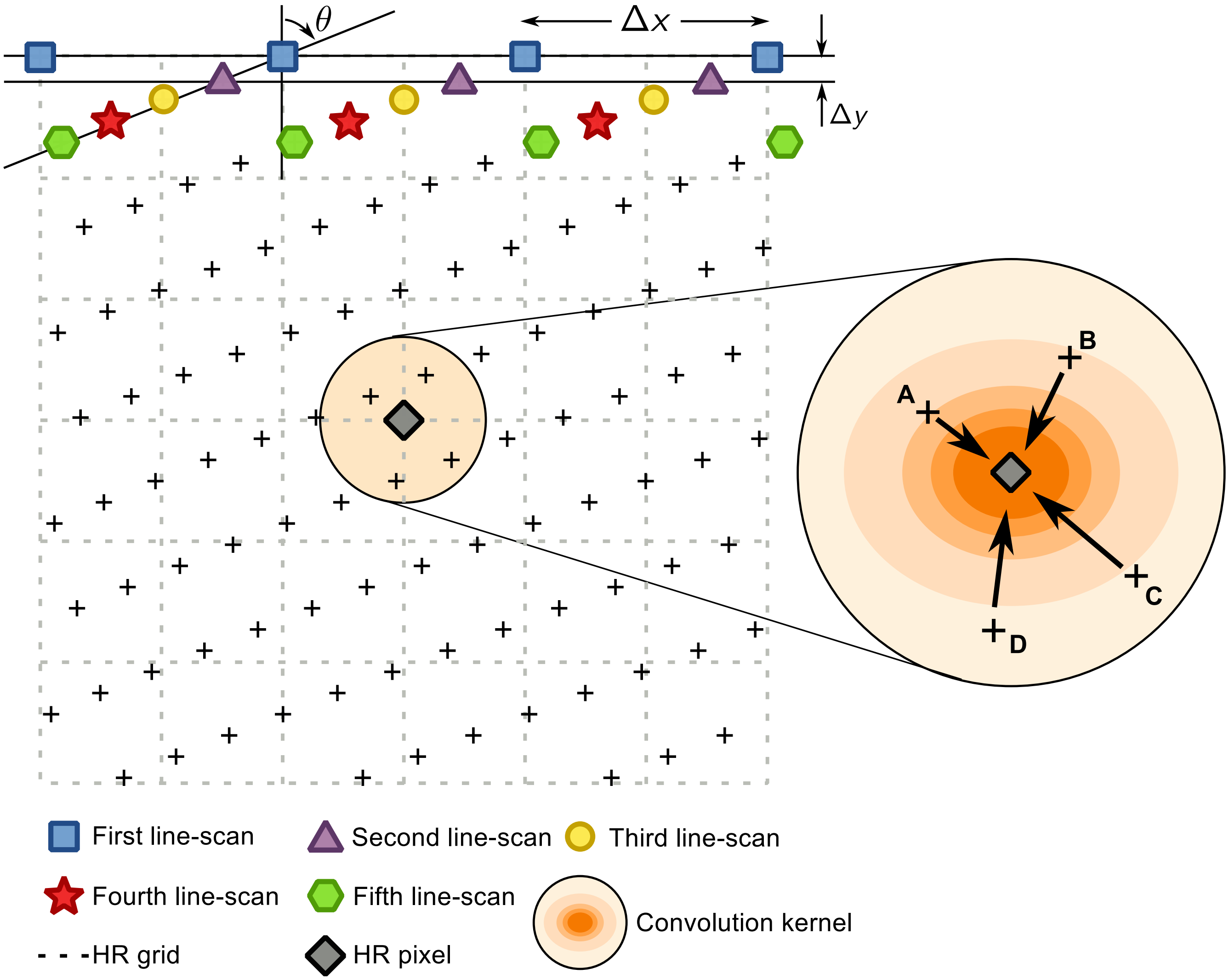

Figure 3. **Illustration of the denoised non-uniform interpolation of the pixel-SR time-stretch image reconstruction algorithm.**

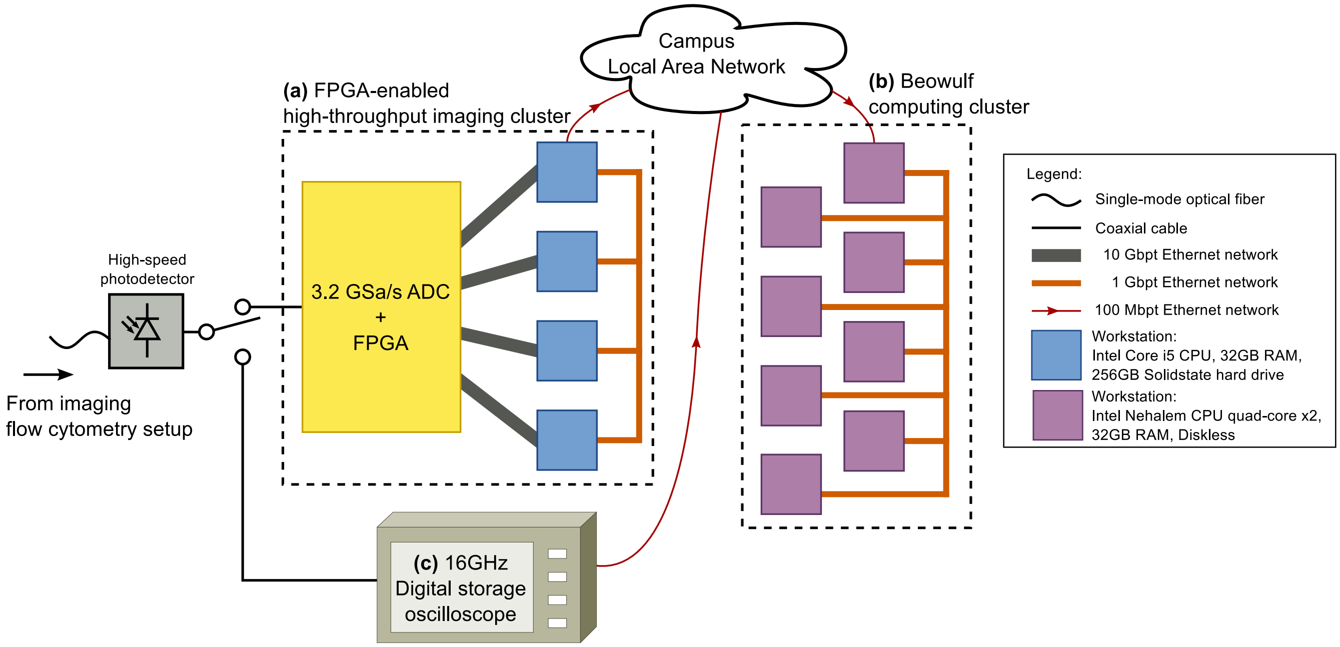

Figure 4. Computing architecture for digital acquisition and analysis of time-stretch image signal. (a) digitizer with field-programmable gate array capable of giga-pixel time-stretch imaging; (b) high-performance computing cluster for parallel time-stretch image restoration and analysis; (c) high-end oscilloscope for image resolution comparison.

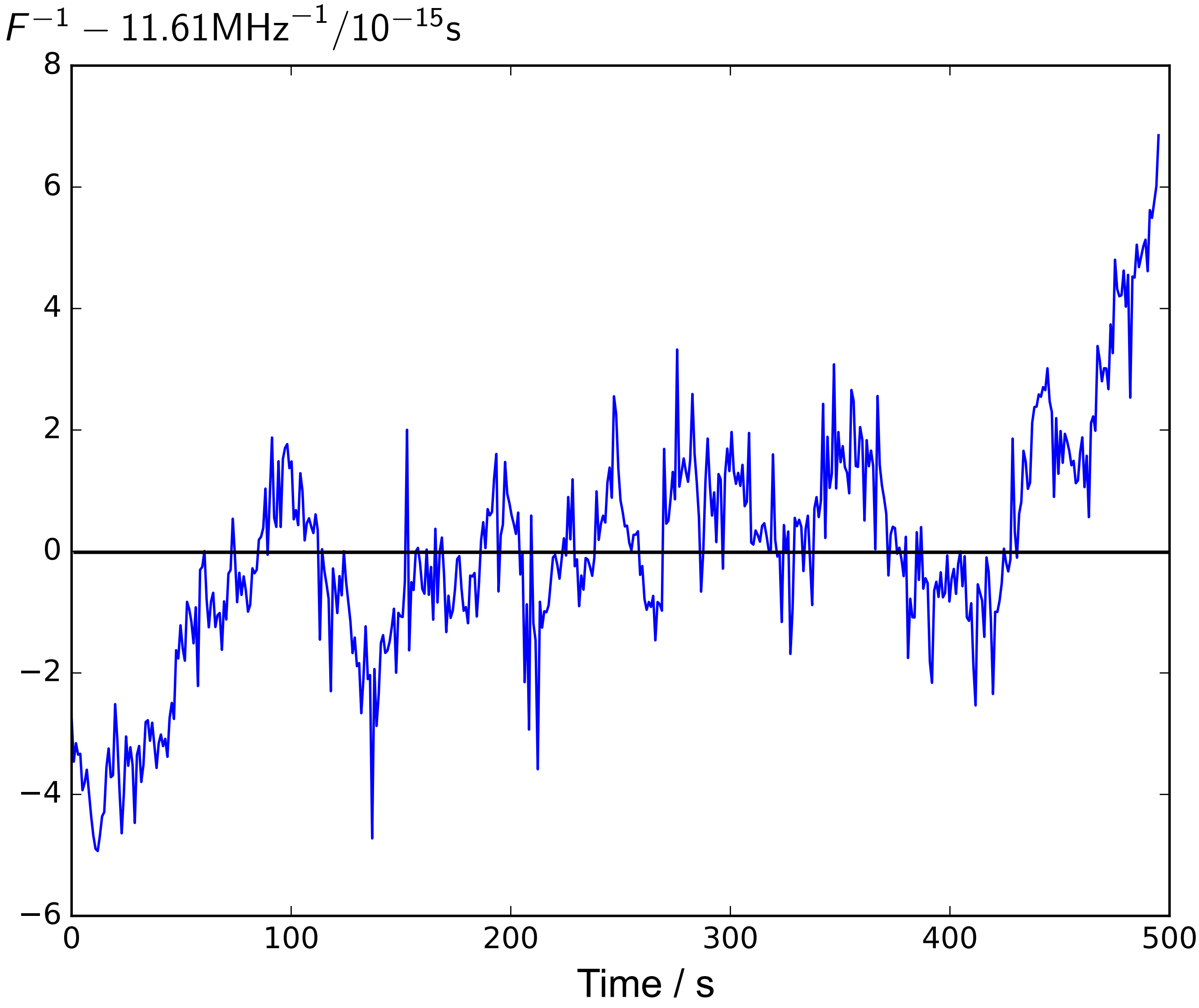

Figure 5. Timing jitter and long-term stability of the home-built mode-locked laser. The magnitude of the time-jitter is in the order of 10^{-15} s, i.e. 0.001% of the sampling interval of the commercial-grade ADCs.
